# Bell's Palsy: A Review

**DOI:** 10.7759/cureus.30186

**Published:** 2022-10-11

**Authors:** Awantika Singh, Prasad Deshmukh

**Affiliations:** 1 Department of Otorhinolaryngology, Jawaharlal Nehru Medical College, Datta Meghe Institute of Medical Sciences, Wardha, IND

**Keywords:** electromyography, nerve excitability test, acyclovir, corticosteroids, lower motor neuron palsy, facial nerve, bell's palsy

## Abstract

Bell's palsy, also known as “acute facial palsy of unknown cause”, is a common cranial neuropathy leading to facial muscle paresis or complete paralysis characteristically on one side, occurring suddenly and may progress over 48 hours. It results from facial nerve dysfunction due to trauma or inflammation of the 7^th^ cranial nerve or facial nerve or its branches along its course, primarily in the bony canal. Both sexes are equally affected, and though no age is immune, its incidence rises with increasing age. The risk is high in diabetics, hypertensives, women who are pregnant, obese, and people with upper respiratory tract infections. It is considered chiefly idiopathic and is diagnosed by the exclusion of other causes. Bell's palsy can cause physical and psychological complications and negatively impact patients and their relatives. Thus, early diagnosis and quick cause determination are prime roles in proper treatment. However, the exact etiology of Bell's palsy is unknown, affecting its treatment. Still, determining probable causative and risk factors is critical for employing a targeted treatment approach and requires a comprehensive examination and a complete history. Although the majority of patients recover spontaneously in less than three weeks even if they are not treated. But there is always a risk of residual paresis after treatment or recovery, which may require medical help. This review aims to furnish the most thorough understanding of Bell's palsy, focusing on anatomy, etiology, clinical features, diagnosis, clinical consequences,and preferred therapy approaches.

## Introduction and background

Introduction

A human’s face is a very integral part of his identity and uniqueness. Facial expressions play a crucial role in expressing emotions and in social interactions, thus any defect in facial muscle control, besides physical disability creates social and psychological distress [1]. Bell's palsy is an acute-onset peripheral facial neuropathy and is one of the most frequent causes of lower motor neuron facial paralysis. Bell’s palsy is implicated in 60-75 percent of all cases of facial paralysis. Every year, 7-40 cases occur per 100,000 people, the prevalence being similar in both genders. The cause remains idiopathic but strongly associated with certain viral infections, resulting in nerve inflammation causing focal edema, demyelination, and ischemia. According to various studies, certain risk factors like increased blood sugar [2], uncontrolled blood pressure, severe pre-eclampsia [3], migraine [4], and radiation exposure [5] aid in the pathologic processes and make an individual more prone to palsy. The weakness could be complete or partial and may be associated with numbness, mild pain, enhanced sound sensitivity, and alteration in taste. The diagnosis is one of exclusion and is primarily determined based on physical examination. 

An introductory study of the neuroanatomy of the nerve can help discern the difference between a central and a peripheral lesion. Because the management differs with different etiology, this distinction is critical. With the doubtful utility of antivirals, these are primarily recommended in combination with corticosteroids. Patients showing signs of improvement within the first three weeks of the development of symptoms have a higher chance of complete recovery; therefore, the sooner the healing begins, the lesser the chance of developing complications and residual paraesthesias. Four to 14 percent of patients may experience recurrence, with 36 percent suffering from palsy on the same side [6].

Material and methodology 

In this narrative review, we retrieved the literature on Bell’s palsy from PubMed, Web MD, Medscape, Google Scholar, Cochrane Database of systematic review, and some standard textbooks. While browsing through various databases, we used the advanced search option, and all relevant articles from 2011 to 2022 were considered. We used keywords and phrases in multiple combinations: 'Bell's palsy, 'facial palsy', 'Bell's phenomenon, and idiopathic facial paralysis'. The reference list of relevant publications was also employed as a potential source of information. If older studies (10 years or older) were deemed essential to conclude, we incorporated them in the discussion section to make this review more inclusive. No attempts to discover unpublished data were constructed.

Background

Bell's palsy is named after Sir Charles Bell (1774-1842). Although he was the first to present the anatomical basis of Bell's palsy, recent research has revealed that other European practitioners contributed earlier clinical descriptions and accounts of the seventh peripheral cranial nerve palsy [7]. The first case report of idiopathic facial paralysis is believed to have been published by an 18th-century medicine professor, Nicolaus Friedrich, in Wurzburg. The case report described three middle-aged men with similar episodes of unilateral facial paralysis, which was subacute or acute in origin and gradually bettered in a few weeks to months. Later, Charles Bell studied facial nerve function in animals. He encountered several unilateral facial nerve paralysis cases during his surgical practice in London. His most well-known and widely cited case of facial palsy was posted in 1828, in which he presented a matter of a man hit by a bull and the resulting injury caused perpetual facial nerve paralysis [8].

## Review

Anatomical perspective

For a better comprehension of the etiopathogenesis of Bell's palsy, basic knowledge about the course and innervations of the facial nerve is required. The facial nerve has three nuclei: motor, sensory and parasympathetic nuclei. The course of the facial nerve can be divided into six segments. The first segment is the intracranial segment which comprises of facial nerve's motor nucleus located in the pons from where the motor fibers originate, hook around the abducens nerve nucleus, and are joined by the intermediate nerve which carries sensory and parasympathetic components. Further, this mixed nerve passes through the posterior cranial fossa and enters the bony facial canal (fallopian canal) through the anterior superior quadrant of the internal acoustic meatus. This is known as the meatal or canalicular segment. Inside the inner ear, the facial nerve passes in the fallopian canal in between the cochlea and vestibule and then bends posteriorly at the geniculate ganglion (first genu). This segment is the shortest and narrowest and is most prone to inflammation and ischemia. It is known as the labyrinthine segment. The labyrinthine segment extends and forms the tympanic segment in the middle ear, takes another turn just distal to the pyramidal eminence (second genu), and passes vertically downwards as the mastoid segment. The bony fallopian canal in many cases can be dehiscent in some areas and thus more susceptible to damage. The mastoid segment starts from the second genu, gives off its branches, and ends at the stylomastoid foramen forming the extratemporal segment. It further passes in between the superficial and deep lobes of the parotid gland and finally terminates into five branches at the anterior border of the gland [8,9].

The facial nerve gives efferent motor supply to all the muscles of facial expression, the stapedius, the posterior belly of the digastric muscles, and parasympathetic and sensory fibers [10]. These parasympathetic fibers supply the submandibular and the lacrimal glands via the chorda tympani and the greater superficial petrosal nerve, respectively [8]. Therefore all these fibers and structures are susceptible to paralysis in case of any damage to the facial nerve. Extended and convoluted pathways and presence in a narrow bony canal make the 7th cranial nerve more prone to paralysis than all other nerves in the body [11].

Etiopathogenesis

Although the precise pathogenesis of Bell’s palsy is not known and considered idiopathic, specific immune, ischemic and hereditary factors strongly correlate with its etiology. Based on recent reports, the reactivation of dormant herpes virus in the geniculate ganglion and its migration to the facial nerve has been considered to be vital in causation [8,12]. Herpes zoster virus (HZV) and herpes simplex virus (HSV) are human neurotropic alpha herpes viruses and are most commonly involved [13]. These may remain latent life-long in the ganglia [14]. HZV virus is considered more aggressive as it spreads across the nerve via satellite cells. Usually, herpes simplex is involved in causing cold sores and genital herpes, whereas herpes zoster is causative of chickenpox and shingles. The infection is said to be latent when there is no active viral replication, but in the presence of antibodies or immunodeficient states, nerve damage and inflammation of the facial nerve may occur, resulting in its further compression due to its fact in a narrow bony canal. Other viruses known to be involved in the causation of Bell's palsy are Epstein Barr virus causing infectious mononucleosis, cytomegalovirus, adenovirus, mumps virus, influenza B, etc. [15].

Vascular ischemia may be primary, secondary, or tertiary. Primary ischemic neuropathy, which causes inflammation of the afflicted nerve, is more prone to take place in specific clinical circumstances, such as diabetes mellitus [10]. It is usually induced by cold or emotional stress. Even though the facial nerve has good vascularity and a tough epineurium, vasospasms can cause a reduction in blood flow and acute inflammation, resulting in primary ischemic neuritis, which is uncommon [16]. It may be followed by secondary ischemia, which further aggravates nerve damage by causing increased capillary permeability leading to fluid accumulation, edema, and thus nerve compression [17]. In about 4-14% of individuals, hereditary predisposition narrows the fallopian canal. This genetic component is mostly autosomal dominant and put the nerve at additional risk of early compression with even the slightest edema [18].

Clinical presentation

**Figure 1:**
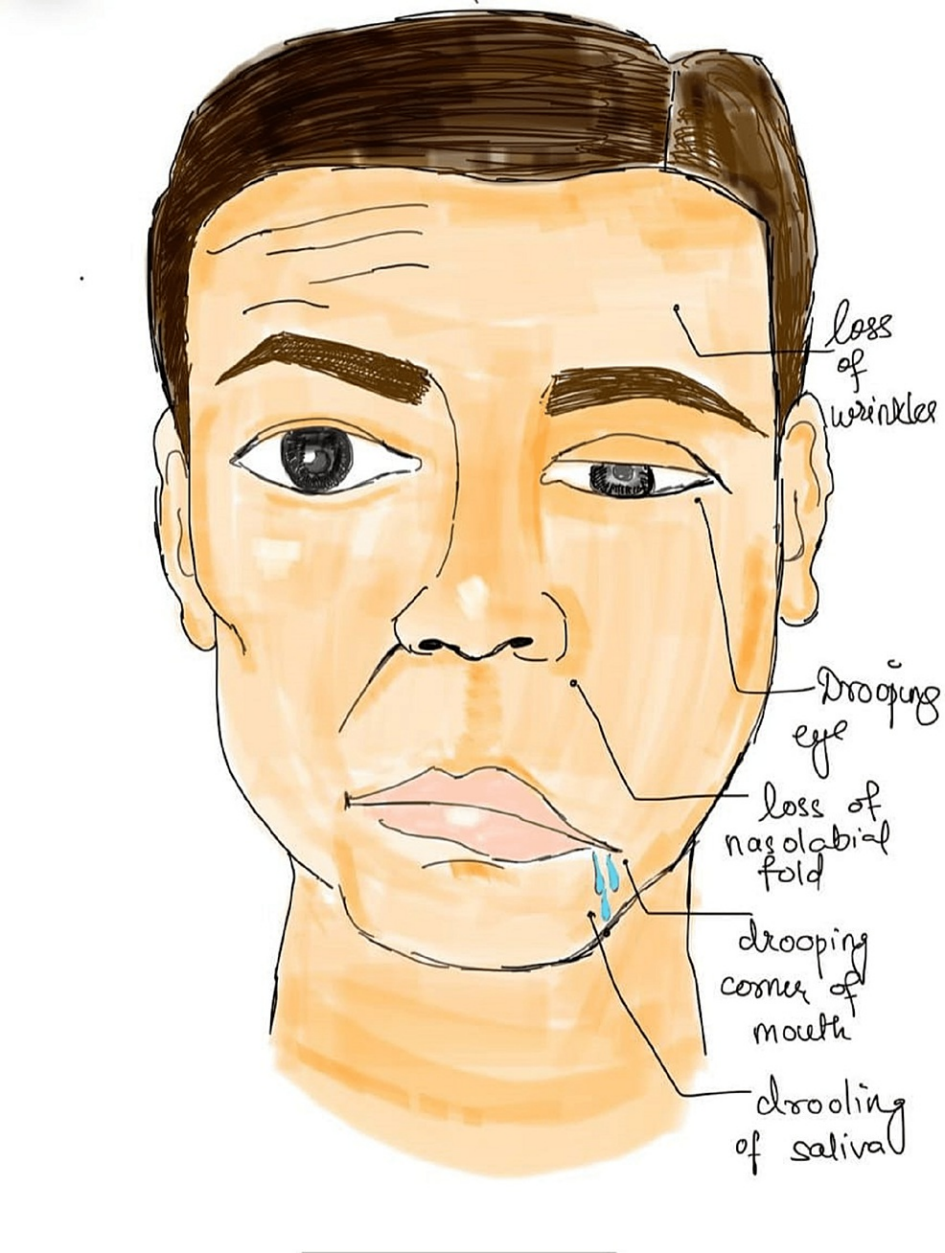
Depiction of clinical features of a patient with Bell's Palsy

The occurrence of symptoms is sudden, and the severity reaches a peak within 48 to 72 hours and ranges from mild fatigue to severe paralysis of facial muscles of the ipsilateral side. Symptoms of Bell's palsy include the inability to blink or close the eye, ruck up the lips or raise the mouth corner and shows features like drooping of half of the face, ipsilateral sagging of the eyebrow, nasolabial fold flattening, ipsilateral pain around the ear or hearing impairment, dry eye or dry mouth as shown in Figure 1 [19].

Other symptoms include hyperacusis caused by nerve fiber breakdown in the stapedius muscle, alterations in taste, and dry eyes caused by parasympathetic affliction. Some patients report facial paresthesia, which is usually motor symptoms misinterpreted as sensory alteration and present with sensory or hearing loss [20].

Clinical examination and diagnosis

A thorough neurologic and general examination, including ear examination, ophthalmological examination, and consideration of the skin and parotid gland, should be included in the clinical study [21]. Herpes zoster may be suspected if there are blisters or scabbing around the ear known as Ramsay-Hunt syndrome which can result in hearing loss and also facial nerve palsy. Observing the patient during the interview may reveal subtle symptoms of weakness and provide ancillary information [7].

A methodical approach to the evaluation of such patients is imperative. Examiner has to observe for facial expressions and test facial movements like wrinkling of the forehead (temporal branch), ability to close eyes tightly, puffing of face (mandibular), symmetry of smile, screwing up eyes (zygomatic branch), wrinkling nose (buccal). The degree and prognosis of facial nerve palsy can be evaluated by the House-Brackmann grading system. As shown in Table 1, it has six grades, with Grade 1 being no paralysis and Grade 6 meaning complete paralysis [22].

**Table 1:** House- Brackmann facial nerve grading system

Grade	Description	Characteristics
1	Normal	Normal facial function in all areas
2	Mild dysfunction	Slight weakness noticeable on close inspection; may have very slight synkinesis
3	Moderate dysfunction	Obvious, but not disfiguring, difference between two sides; noticeable, but not severe, synkinesis, contracture, or hemifacial spasm; complete eye closure with effort
4	Moderately severe dysfunction	Obvious weakness or disfiguring asymmetry; normal symmetry and tone at rest; incomplete eye closure
5	Severe dysfunction	Only barely perceptible motion; asymmetry at rest
6	Total paralysis	No movement

Wrinkling on the forehead on the ipsilateral side is absent or appears asymmetric while raising the eyebrows. The ipsilateral eye shows partial closure and may remain slightly open when the patient attempts to close his eyes, and the involved eyelid may slightly lag when the patient blinks. The examiner can demonstrate Bell's phenomenon by attempting to open the eyelids of the patient while he is asked to shut the lids tightly. The eyes, in such a case, deviate upwards and laterally. This procedure can also assess the strength of the orbicularis oculi muscle. Careful ear examination for cholesteatoma, acute suppurative otitis media, chronic suppurative otitis media, malignant otitis media, and any other signs of middle ear disease is required. Red chorda tympani (vascular flaring of the tympanometry area) is seen in Bell's palsy. Various audiometric tests like pure tone audiometry, speech audiometry, brainstem evoked response audiometry, and special tests can be performed to rule out cochlear and retrocochlear lesions.

To know which segment of the facial nerve is involved, physicians should perform prognostic tests like tearing, salivation, taste, and stapedial reflex, while electrodiagnostic tests will reveal the depth of damage. The diagnosis of Bell's palsy is mainly clinical, and it is made by ruling out alternative causes of unilateral facial paralysis [23]. Electrodiagnostic tests performed within 14 days of commencement may provide prognostic information. The majority of cases of facial paralysis are caused by some other ailment that mimics Bell's palsy, such as a central lesion like stroke or demyelinating disease, cholesteatoma, parotid gland tumor, middle ear infections, Lyme disease, diabetes, granulomatous disease, Ramsay-Hunt syndrome, trauma, and Guillain-Barré syndrome [20,24,25]. 

A careful history, complete head and neck, and otological examination are paramount, along with radiologic studies, blood tests such as peripheral smear, total count, blood sugar, sedimentation rate, and serology. Whether or not the forehead muscle is involved in paralysis can help in clinically determining whether the palsy of the facial nerve is due to central causes like stroke or is peripheral. The upper part of the facial nerve nucleus, which supplies the frontalis muscle, receives fibers from both the cerebral hemispheres. In contrast, the lower part of the nucleus supplying the lower and middle face gets only crossed fibers from one hemisphere, so if the lower and central portion of the face is paralyzed, but the function of the frontalis is preserved, the lesion must be supranuclear. The absence of forehead wrinkles, incomplete closure of eyelids, sagging of eyebrows, flattened nasolabial folds, and stooping of the corner of the mouth are all common symptoms of peripheral facial nerve palsy.

Nerve excitability tests are done regularly to monitor nerve degeneration. They record the least value of electrical stimulus, which can produce a visible muscle contraction, thus helping determine the excitation threshold. The excitation threshold of the involved side is then compared with that of the uninvolved side, and if the difference is more than 3.5 mA, the prognosis is poor. Imaging and laboratory tests are more reliable in patients showing no betterment in symptoms even after three weeks of therapy or those with recurrence [8]. Intensification of nerve in MRI when correlated with history and examination is diagnostic of Bell’s palsy. Intensification of tympanic and vertical segments may occur in a normal person, but specifically, labyrinthine segment intensification is seen in Bell's palsy [26].

Motor nerve conduction studies and electromyography of the facial nerve can aid in the patient's pharmacotherapeutic and surgical management by assessing the nerve's viability and functioning [8]. Such electrodiagnostic tests can measure the provoked action potentials in involved muscles and thus helps in estimating the extent of axonal damage. Patients having axonal degeneration greater than 90 percent can be managed by surgical decompression. In contrast, those with a lower amount of axonal degeneration do not require surgical interventions and have a favorable prognosis.

Treatment

Most patients usually have a good prognosis. According to The Copenhagen Facial Nerve Study, the majority of patients recover completely, around 13 percent suffer slight paresis and 4 to 5 percent are left with significant facial dysfunction [27]. As spontaneous recovery is usual, the treatment is still controversial, but medical treatment and therapies help relieve symptoms and hasten recovery.

Prednisone and other oral corticosteroids reduce nerve swelling and may speed up the recovery of facial actions and expressions. These medications are most efficacious when started within 48 hours of the onset of symptoms [12]. When taken with corticosteroids, antiviral drugs such as acyclovir for herpes are known to hasten the healing. This combination has a short treatment period and is cost-effective thus is very often recommended unless contraindicated [28]. Ear pain can be relieved with the use of analgesics.

**Figure 2:**
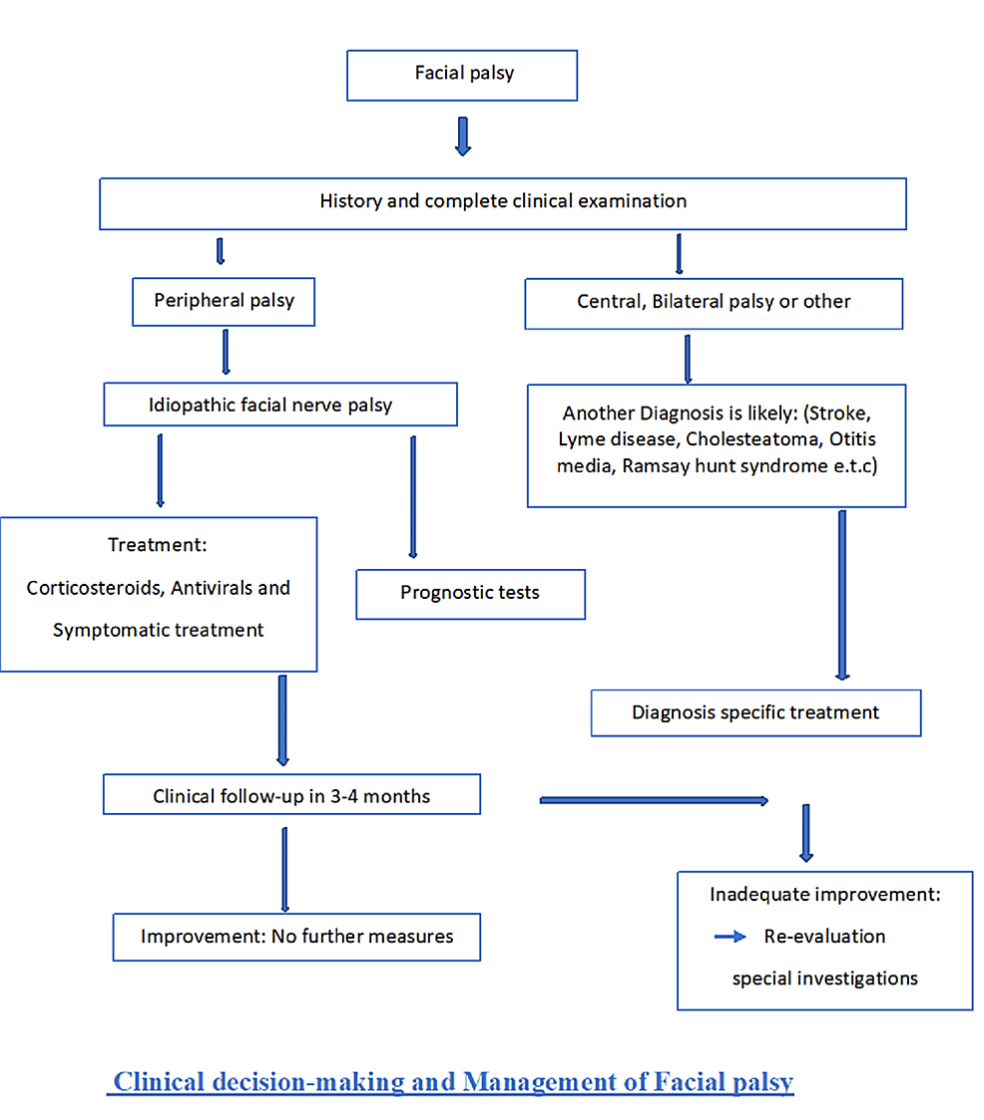
Clinical decision-making and management of the patient with Bell's palsy

Oral corticosteroids, such as prednisone, reduce nerve swelling and might improve facial motions and expressions faster [29]. This treatment is most efficacious when it is started within 48 hours of noticing symptoms [30]. The suggested dose of prednisone is 60 mg orally once a day for five days which is then tapered to 10 mg per day [31]. Antivirals like acyclovir (Zovirax®) can be initiated at a dose of 400 mg orally five times a day and continued for 10 days in case of associated herpes infection. Figure 2 describes the complete management of a patient with Bell's palsy according to the course of the disease.

Various nonpharmacologic measures like physiotherapy, including facial exercises, neuromuscular retraining, and acupuncture, are also implied in treating Bell's palsy and are reported to hasten recovery [32]. As in Bell's palsy, the patient’s ability to close or blink their eyes is impaired, and the affected eye is exposed to dryness and risk of potential injury. Eye pads, goggles, or the insertion of a small spring can ensure protection, and lubricating eye drops and artificial tears during the daytime can control dryness [33]. Muscle weakness and associated facial asymmetry in Bell's palsy can cause difficulty in swallowing, slurring of speech, difficulty in drinking and eating, etc. Such patients can be given occupational and speech therapy, which helps improve speech clarity, lower problems associated with dysphagia, and reduce social awkwardness.

If paralysis does not improve in six-eight weeks, facial nerve decompression can be tried by an opening sheath or eggshell bone removal. In severe cases, for those who don't recover, functional facial plastic surgery procedures can be an option to correct facial asymmetry and help with eyelid closure. Laser acupuncture is also tried in parts of Asia for patients with acute bell's palsy, though its role in chronic Bell's palsy is still questionable [34]. It is a painless, non-invasive mode of treatment used in various inflammatory painful conditions and can be an effective modality in patients with inadequate recovery from Bell's palsy [35].

## Conclusions

Bell's palsy is an ipsilateral, idiopathic, and acute lower motor neuron paralysis of the seventh cranial nerve that causes weakening of the platysma and facial muscles and significantly impacts the patient's appearance and the standard of living and psychosocial well-being. Symptoms begin with mild weakness in facial muscles without any neurologic abnormalities and peak in the first week and then steadily diminish over three weeks to three months even without any medical treatment but may result in various complications and leave the patient with varying degrees of residual paralysis if timely diagnosis and interventions are not taken. It can affect any age and has an equal effect on both genders though its incidence peaks in the 40s and often occurs in people with diabetes.

Diagnosis is one of exclusion and requires a vigilant history and thorough clinical examination. If stipulated by medical history or risk factors, testing for Lyme disease and diabetes can be suggested. Incomplete closure of lids with resultant dry eye, dysphagia, and slurred speech are common short-term complications. An uncommon long-term complication is contractures and the permanent weakening of facial muscles. Although most patients undergo spontaneous recovery, treatment with a short course of valacyclovir or acyclovir and a tapering dose of prednisone, started within three days of the appearance of symptoms, is considered to shorten the time and chance of complete recovery.
